# Rehabilitative game-based system for enhancing physical and cognitive abilities of neurological disorders

**DOI:** 10.1007/s11571-025-10229-x

**Published:** 2025-03-10

**Authors:** Neven Saleh, Ahmed M. Salaheldin, Maged Badawi, Ahmed El-Bialy

**Affiliations:** 1 Biomedical Engineering Department, Faculty of Engineering and Technology, Future University in Egypt, New Cairo, Egypt; 2 Biomedical Engineering and Systems Department, Faculty of Engineering, Cairo University, Giza, Egypt; 3 Medical Engineering, Technical Faculty, University of Erlangen-Nürnberg, Erlangen- Nuremberg, Germany

**Keywords:** Neurological disorders, VR, Rehabilitation, Game-based system, Regression analysis, Physiotherapist

## Abstract

Neurological disorders affect the nervous system and can impair physical, cognitive, or emotional functions. They often result in challenges such as movement difficulties and the inability to perform daily activities. Common conditions include stroke, traumatic brain injury, and cerebral palsy. Physical therapy is a common approach to managing these disorders. Recently, virtual reality (VR), a technology that creates interactive, simulated environments, has been used in rehabilitation. This study presents a rehabilitative game-based system to improve patients’ movements and cognitive abilities. Six games were designed using the Unity platform, namely, “Piano,” “Connect,” “Drag & Drop,” “Little Intelligent,” “Memory,” and “Hack & Slash.” The Oculus Quest 2 VR headset was used to simulate the virtual environment for gaming. A mobile application called “Recover Me” was created to facilitate communication between patients and physiotherapists. A score index was generated for each patient, indicating the performance. It enables monitoring and assessment of the patients, leading to customizing the treatment plan based on progress. The study proposed simulating monitoring and evaluation of the patients by training an artificial neural network model to predict scores for the developed games and consequently indicate the patient’s actual status. A dataset of 50 patients with different injuries was used. Results indicate patient satisfaction with gaming and enjoyment. Moreover, a regression analysis was performed to detect the progress level of each patient, indicating that 60% of the tested patients had improved. A low-cost VR game-based system has proven effective in rehabilitating neurological disorders.

## Background

Neurological disorders encompass a wide range of diseases that significantly impact physical and cognitive abilities (Yu et al*.*, 2024)*. Parkinson’s disease* is characterized by progressive motor symptoms such as tremors, rigidity, and bradykinesia, which directly affect movement. *Traumatic brain injury (TBI)* often results in impaired motor functions, coordination issues, and challenges in cognitive processing. *Stroke* leads to sudden loss of motor control and muscle weakness, typically on one side of the body, along with potential cognitive impairments. *Alzheimer’s disease* primarily affects memory, reasoning, and problem-solving abilities*. Cerebral palsy* causes permanent movement disorders, often accompanied by muscle stiffness and coordination difficulties (Daoud et al. 2020; Wang et al. 2020). Due to the characteristics of these conditions, motion impairment is the most critical disability to monitor for rehabilitation. Additionally, cognitive dysfunctions frequently accompany these disorders.

Traditionally, rehabilitative care refers to the practice of exercises that help patients engage with their surroundings. Various regimes are presented for neurological disorder rehabilitation, including sensor-based paradigms, robotic systems, and virtual reality applications (Song et al. 2019). However, all different systems are characterized by repetitive exercises for long periods of time, leading to patient boredom and a lack of motivation (Sinpithakkul et al*.* 2018; Tamayo-Serrano et al. 2020).

Recently, very promising regimes such as virtual reality (VR) and augmented reality (AR) game-based systems have been meticulously considered in the rehabilitation protocols (Lange et al. 2012; Pham et al*.* 2015). According to (Pham et al. 2015; Tamayo-Serrano et al. 2020; Wang et al. 2020), these programmes go beyond their treatment purposes because they can also provide entertainment, training, and education. Game-based rehabilitation systems have proven their robustness in Parkinson’s disease (Wang et al. 2020), hand rehabilitation (Potigutsai and Sornil 2021), cerebral palsy (Sinpithakkul et al*.* 2018; Daoud et al. 2020; Yin et al. 2024), stroke (Pham et al*.* 2015; Song et al. 2019; Tamayo-Serrano et al. 2020), visual perceptual dysfunction (Wuang et al. 2021), tremor (Kantu et al. 2024), etc. Regardless of the adopted protocol for physical therapy, any rehabilitation system takes place in a hospital, clinic, or at home.

A home-based rehabilitation regime is deemed preferable for an array of reasons, including low cost, comfort, reduced labor intensity, less reliance on assistance, and proximity to caregivers (Song et al. 2019; Daoud et al. 2020), making a game-based rehabilitation system an attractive method for the sustainable practice of physical therapy. These reasons led us to develop a home-based, game-driven rehabilitation system using VR technology. The system includes six games designed to address various neurological disorders, such as stroke, traumatic brain injury, and cerebral palsy. Based on the gamification approach, patients engage with the games while being monitored by a physiotherapist through a dedicated mobile application. Remote monitoring enables the customization of therapy plans to meet individual patient needs without requiring physical intervention.

The games are designed to accommodate different levels of patient abilities. An artificial intelligence (AI) driven solution was developed to evaluate the effectiveness of the rehabilitation approach. This model aims to estimate patient performance and track treatment progress, supporting the physiotherapist in monitoring each patient’s improvement. Overall, the proposed system offers a comprehensive solution for the rehabilitation of individuals with neurological disorders.

Contributions of the study can be summarized as follows: (1) targeting various neurological disorders survivors, (2) designing a series of six new games for rehabilitation, (3) developing mobile application that permits monitoring and assessing patients remotely without a physiotherapist’s existence, (4) developing an AI driven solution to assess patient performance and anticipate the progress of treatment (5) presenting a low-cost solution that can be adapted to different disabilities, and (6) providing an alarming system in risky situations.

The structure of the article is organized as follows: the relevant studies are discussed in the Related Work section. A description of the designed games and AI model is provided in the Materials and Methods section. The results of the applications of the designed games and AI model are presented in the Results section. In the Discussion section, we explain the outcomes of the designed system and compare our results with those of other related studies. Lastly, conclusions of the study and guides for future work are given in the Conclusions section.

## Related works

Many studies have been conducted to present game-based solutions for rehabilitation. In 2021, Potigutsai and Sornil developed a game-based system for improving hand movement using deep learning techniques. Through the YOLO V3 algorithm, hand and fingertip detections were identified. Three metrics were proposed among the participants to evaluate the designed game by measuring enjoyment, convenience, and effectiveness (Potigutsai and Sornil 2021). Stroke rehabilitation was addressed in (Song et al. 2019) by using a cell phone AR system. Three serious games were designed to regain upper limb functions, cognitive ability, and improve the mental state of pro stock patients. The three games were named “Stroop Game,” “Pyramid Reach,” and “Add vs. Sub”. The system was validated by testing it on eight post stroke survivors. In a similar context, a rehabilitative, serious game-based AR system was developed for Parkinson’s disease (Wang et al. 2020), where improvements in gait, balance, and turning abilities were observed in the investigated subjects.

Enhancing patient motivation and engagement was tackled by Tamayo-Serrano et al. (2020). Based on two different design approaches, a game-based rehabilitation system was developed for recovering stroke patients at home. First, the games were chosen and modified in the therapy program according to the therapeutic objectives. Second, hospital-use physiotherapy exercises were selected and virtualized. Six games have been designed to be used by stroke patient at home and at the same time to be validated by the physiotherapist at hospital. The deficiency levels of stroke patients were put in consideration for the designed games. The system was tested by adopting user-centred interface.

Visual perception rehabilitation was addressed in (Wuang et al. 2021). The AR technology was applied to two groups of subjects according to their impairment levels. Two distinctive games were used for each group separately to test the visual perception and the visual outcomes. Consequently, visual-motor skills were reported and tested for improvement. In another context, the VR game-based rehabilitation system was employed for upper extremities (Pham et al. 2015). The system consists of two modules related to the detection of hand motion parameters and game exercise generation. Based on the hand movements, the system maps between the identified game patterns and the rehabilitation requirements. Hand motion was detected by a webcam camera to ensure a low-cost strategy.

Kantu et al. (2024) presented a rehabilitation solution for activities of daily living (ADL). The authors focused on patients with motor impairments and tremors. Six game-based targets for ADL were designed. A novel robotic rehabilitation system called SPINDLE was developed to stimulate ADL tasks in a VR environment. According to the optimal resistance of the subject, the dexterous ADL task is identified. In this way, the system can perform various ADL tasks with 3D movements. The system can stimulate flipping a book, opening a laptop, opening a jar, using a screwdriver, pouring water, and moving a knob.

The impact of using serious video games in physiotherapy on children’s stress levels was the hypothesis of Vural et al. (2024). Two serious video games were designed to recognize stress levels from facial images during physiotherapy sessions. The games “Catch a Pet” and “Leap Ball” were designed for children with dyslexia, obstetric brachial plexus injury, and intellectual problems. Both machine learning and deep learning techniques were applied to classify the facial emotion and recognize stress. The developed system revealed an accuracy of 92% in recognizing facial emotion with stress among investigated children during a serious game-based physiotherapy program.

Based on previous related work, almost all studies have been conducted to alleviate the impact of different disabilities during rehabilitation sessions through video games. Either these games are off-the-shelf games or specially designed games, they demonstrated their effectiveness in disability alleviation. Because VR technology enables an individual to interact with an artificial environment, it emerged as an assistive technology in improving and restoring movement ability. This technology can provide monitoring progress of patients remotely. Therefore, we designed a game-based rehabilitation system that merges restoring physical and cognitive functions simultaneously. Furthermore, in home rehabilitation systems, a physiotherapist is unable to monitor the patient remotely. Therefore, in risky situations, an intervention should occur to prevent catastrophic consequences. With an alert system, serious repercussions with risky situations can be avoided. Thus, the authors proposed to develop a home-based rehabilitation system with six video games based on a VR environment that can be monitored remotely. Using the gamification concept in physiotherapy presents enjoyment, which in turn increases patient satisfaction. Additionally, one challenging issue is to monitor and assess a patient without the existence of a physiotherapist. Due to the scarcity of solutions, we aimed to introduce an AI model to remotely follow up on the rehabilitation progress.

## Materials and methods

**Fig. 1:**
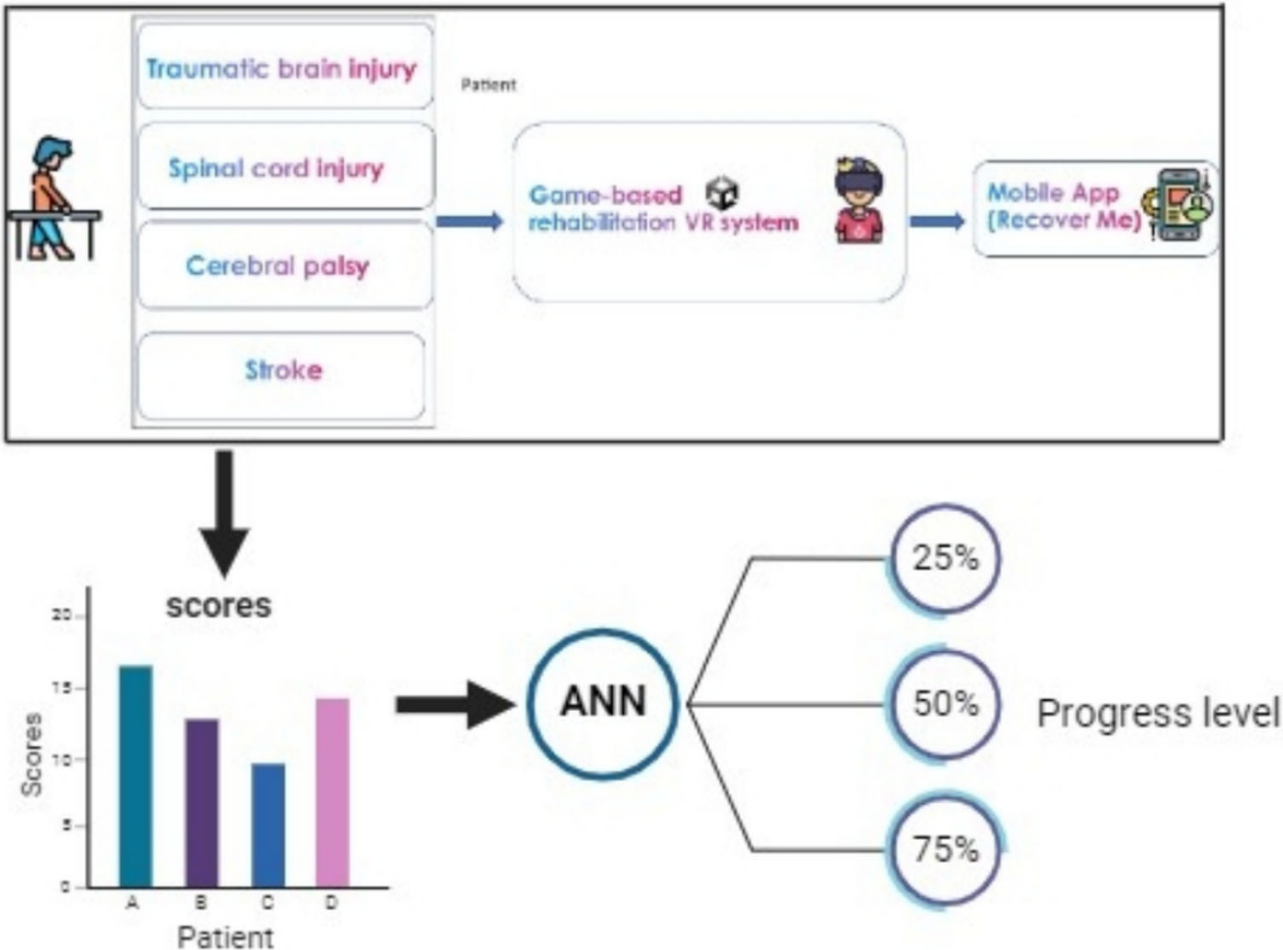
An overview layout of the proposed rehabilitative game-based system for various neurological disorders

In this study, a game-based rehabilitation system is designed to be adopted at home for a range of neurological disabilities, including stroke, traumatic brain injury, spinal cord injury, and cerebral palsy diseases. Six novel video games are designed in VR settings to enhance patient motivation. Moreover, a mobile application, namely “Recover Me,” was developed to interact with a patient and to allow remote monitoring by the physiotherapist. Each game was designed to address a specific disease and improve the physical and/or cognitive abilities of an impaired individual. Therefore, a novel rehabilitative game-based program was designed and implemented based on a VR environment. The plan incorporates a smart adaptive framework combining practicing activities and an alert system to adjust the challenges to each patient’s unique abilities. A general layout of the proposed system is depicted in Fig. 1. To adjust the physiotherapy needs for each disease, several meetings were held with a physiotherapist to indicate the actual requirements for restoring physical activities and cognitive abilities. According to these requirements, game procedures were assigned.

### Game engine architecture

According to the addressed impairments, six novel games were designed to adapt the required physiotherapy activities for each disorder. The goal was to act and assess through the developed system. Each game was designed according to the gathered feedback from the physiotherapist to fulfil the rehabilitation protocol of the patient. Based on ongoing suggestions, each game was tested until the physiotherapist approved it. To promote game functionality, repetitions should be done, and according to the scores gained, the physiotherapist can assess its effectiveness. Therefore, the games enable the patients to engage in physical therapy with enjoyment.

Unity is one of the most widely used game engines, renowned for its versatility and accessibility. It is a cross-platform engine compatible with various systems, including iOS, Android, desktop platforms, virtual reality (VR), and augmented reality (AR), making it ideal for developing applications across multiple environments. Unity supports both 2D and 3D game development, offering significant flexibility in design. Game objects in Unity are created using modular components, with each component controlling a specific aspect of the game, allowing for highly customizable and intuitive development. The engine utilizes the C# programming language, which facilitates efficient scripting. Scripting defines the behavior of game objects, and Unity provides a comprehensive library of variables and functions to support the creation of complex and interactive features (Barczak and Woźniak 2019; Vohera et al. 2021; Singh and Kaur 2022).

### Virtual reality tool

Virtual reality technology has widely emerged in game-based rehabilitation systems. Among a variety of utilized VR headsets, the Oculus Quest 2 is one of the best VR technologies that was developed and launched by Meta Platforms, Inc. The Oculus Quest 2 is a commonly used VR headset, valued for its affordability and ability to create an immersive experience (Raymer et al. 2023). For this research, the Oculus Quest 2 was chosen as the VR device. It includes a headset with integrated tracking sensors and two handheld controllers for user interaction. The device has a memory capacity of 6 GB RAM and storage options up to 256 GB. It features an LCD display with a resolution of 1832 × 1920 pixels per eye, providing clear visuals. Additionally, the headset supports audio functionality, enhancing its usability for gaming and other VR applications (*Oculus Quest 2 Specifications*, 2020).

### Design of games

The Unity engine was used for scripting the designed games with the Firebase backend. Each game was developed based on the desired target of the rehabilitation program. In this study, Unity 2021.3.6f1 version was used to design all games. Six games were created to engage with a VR setting for full immersion in gaming for rehabilitation purposes. The games are “Piano Game,” “Connect Game,” “Drag Drop Game,” “Little Intelligence Game,” “Memory (2D) Game,” and “Hack & Slash Game.”. It is hypothesized that the functional exercises within each game contribute to the recovery of both physical and cognitive abilities. The details and specific characteristics of each game are explored in the following sections.

**Fig. 2:**
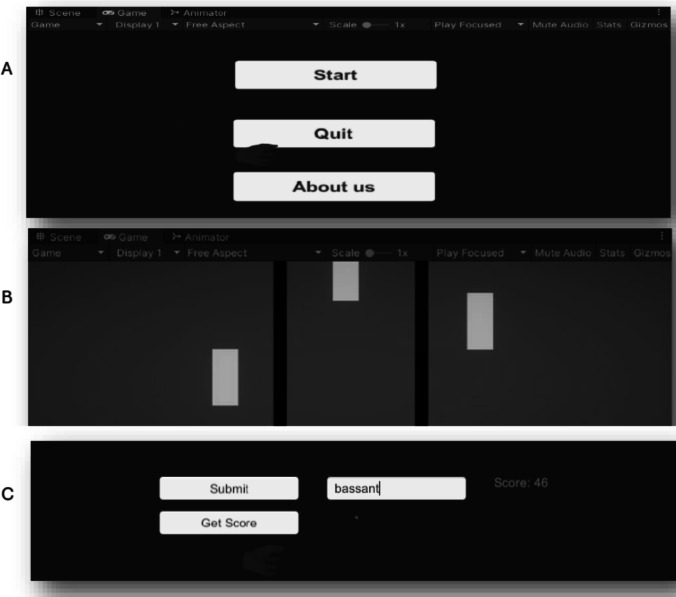
The “Piano Game” showing the scenes of the game; “A” is the start scene, “B” is the gaming scene, and “C” is the ending scene

In “Paino Game (G1),” starting of the gaming scene shows a group of piano tiles. When a patient presses on a tile, a score is recorded. If s/he fails to press a tile, the game is terminated, and a total score of the game appears on the display. The aim of the game was to improve the motor skills of a traumatic brain injury patient and stroke by increasing the strength of the muscles. Moreover, it improves mental skills, such as the memory and recall ability, because the patient can play melodies and favourite songs. The game composed of three scenes as shown in Fig. 2: the start, the gaming, the ending and the submission. The start scene presents three functions; start, quit, and registration. The gaming scene depicts descending piano keys for pressing. In game design, we added the assets, such as scripts and models, based on the playing protocol of the game. Additionally, each scene was created by dragging and dropping appropriate objects from the “Assets” menu.

**Fig. 3:**
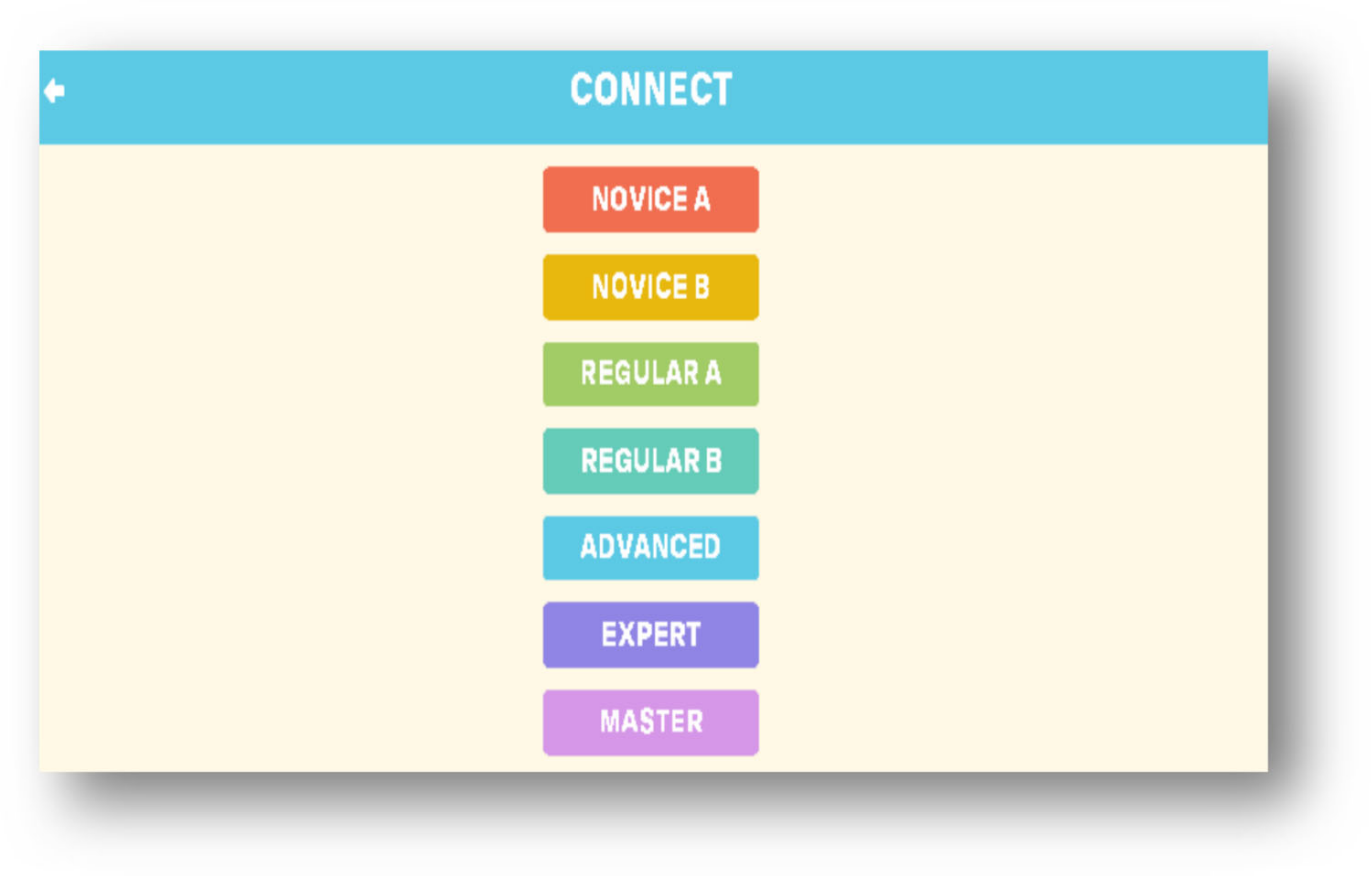
Gaming scene of the “Connect Game,”

The “Connect Game (G2),” contains several levels of playing connection according to the difficulty grade. Seven levels were created, including novice A, novice B, regular A, regular B, advanced, expert, and master, as shown in Fig. 3. When a patient plays at a particular level and wins, the next level appears for continuity. For example, in the level” novice B”, the patient needs to connect the dots of one colour without the intersection of another colour path. The aim of the game was to enhance problem-solving skills, eye-hand coordination, and mental concentration. This game is appropriate for patients with cerebral palsy and stroke. The game starts with pressing the “play” button, then a patient selects the required level based on its difficulty. Each level is composed of 1–50 boxes to play with. By ending the level, the word “win” or “fail” appears, allowing moving to another level or terminating the game.

**Fig. 4:**
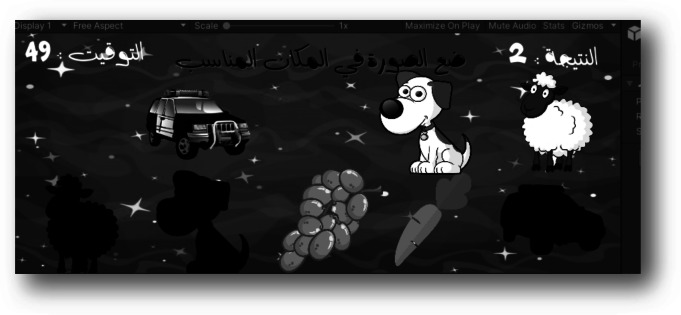
Gaming scene of the “Drag & Drop Game,”

The “Drag & Drop Game (G3),” simulates the process of hand grip strengthening. In this game, the patient opens a group of particular shapes, and s/he is required to drag and drop any shape related to the group. Each time s/he succeeds in doing that, a score is reported. This game improves motor skills, memory, and attention as well. It is helpful to those who are poststroke. Figure 4 depicts a sample of “drag & drop game”. The example shown in Fig. 4 presents dragging and dropping of a dog. The game is composed of two scenes: starting scene and playing scene. The time of dragging and dropping was set for 1 min for each trail.

**Fig. 5:**
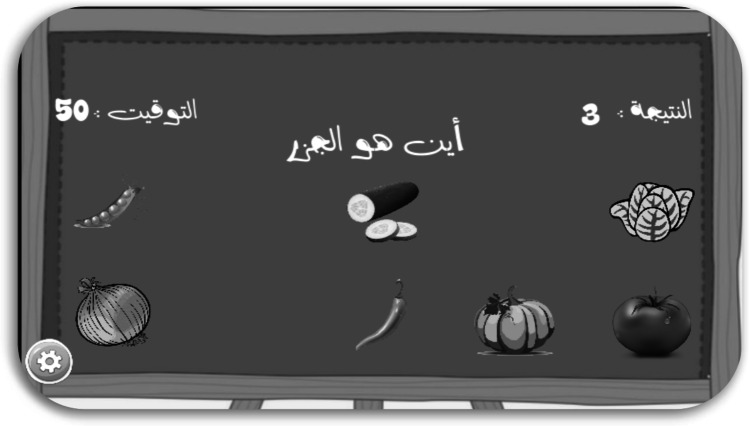
Gaming scene of one level of “Little Intelligent Game”

The “Little Intelligent (G4),” shows how each player can identify a particular object related to a specific class. In this way, five classes were presented in this game, namely fruits, vegetables, geometric shapes, animals, and colours. For instance, when a player opens the vegetable class, as shown in Fig. 5, and the question is, where is the carrot? A countdown timer counts until the correct or incorrect answer is indicated. If the answer is correct, a score is given. This game targets who those with poststroke, cerebral palsy injuries, and traumatic brain injuries. Three scenes were developed for the game, including the start scene, the playing scene with five levels, and the ending scene. In the playing scene, the game opens initially for level 1; otherwise for the previous level of the player.

**Fig. 6:**
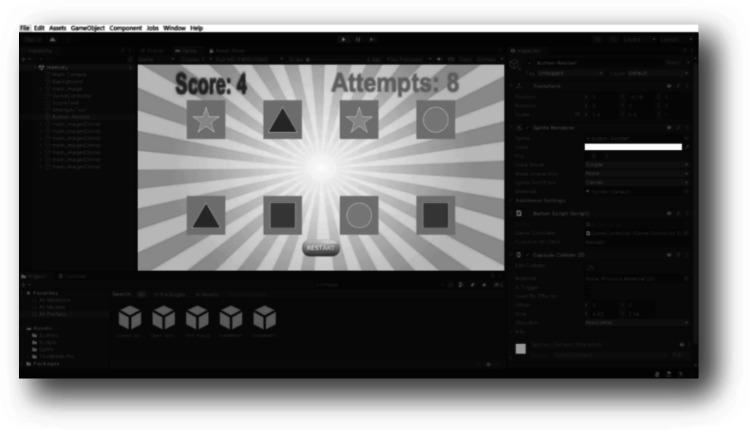
The gaming scene of “Memory Game”

The “Memory Game (G5),” entails activities that improve memory and cognitive abilities. In addition, it can enhance attention and reduce stress because it focuses on improving the mood. In this game, the patient is asked to seek out information about something that relates to an object. For example, naming the shapes and their colours, and giving real examples of an underlined shape. The game allows many attempts to increase the score obtained. Figure 6 shows an example of the “Memory Game”. Alzheimer, cerebral palsy, and stroke patients are the targets of this game. Similarly to the previous games, starting, playing, and ending scenes were created. In the playing scene, the scores and the attempts appeared with the option of restarting the game.

**Fig. 7:**
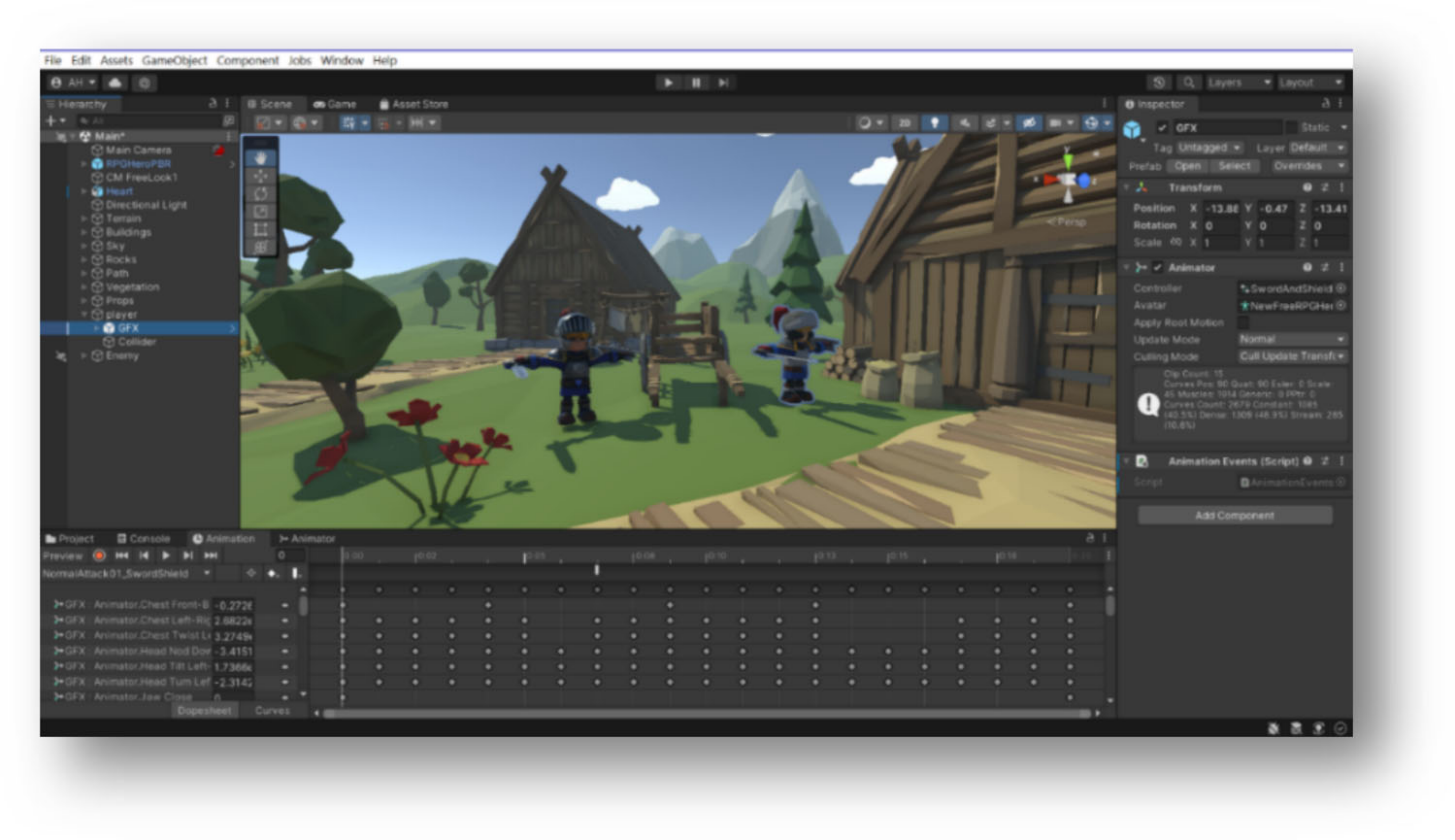
The gaming scene of the “Hack & Slash” game

The sixth game “Hack & Slash (G6),” is mainly designed to regain motor abilities. For physical rehabilitation, this game might be a helpful resource for recovery. Users can conduct exercises and activities that are challenging or unattainable to complete in the actual world by moving and controlling their avatars in a virtual setting. They will have fun participating in an engaging activity that can help them gain strength and mobility. This game necessitates repetitive exercises for effective therapy. For instance, a patient with limited mobility might be encouraged to play a game where s/he must swing a sword or use a bow and arrow to increase the mobility range. Moreover, practicing the “Hack & Slash” allows the player to explore a virtual world that impacts his/her cognitive skills. It develops problem solving and critical thinking skills. A virtual scene of the game is presented in Fig. 7. Considering that the game was designed based on three objects: the environment, the enemy, and the hero.

### Mobile application

**Fig. 8:**
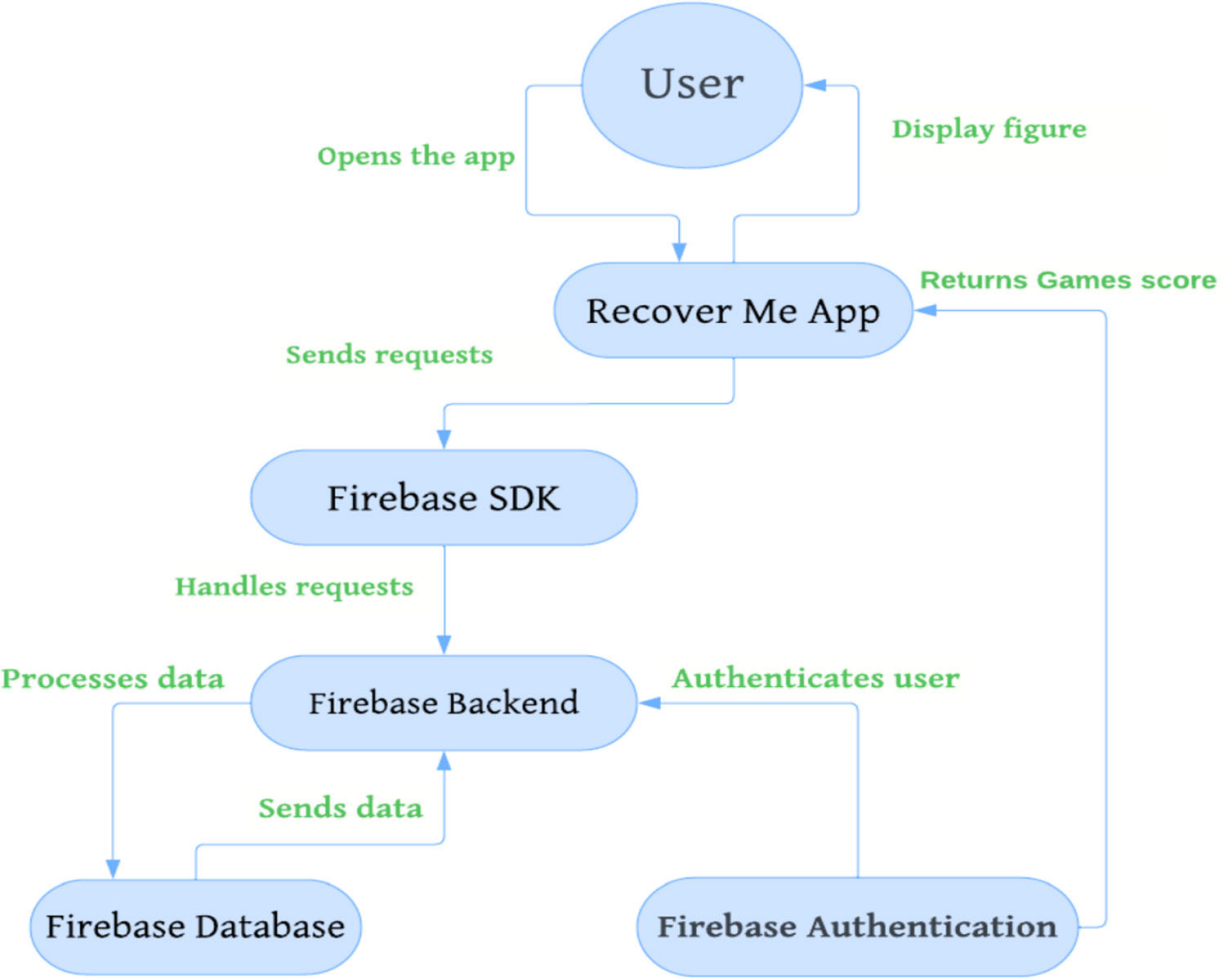
A block diagram of “Recover Me” application showing the flow of data

To communicate with patients who go under the developed rehabilitation system, a mobile application was created. The application permits each patient to select one game out of the six to play and interact with. A score index is given to each game; therefore, it can be assessed by the physiotherapist. Based on the given score, the physiotherapist can estimate the level of improvement for each participant. An Android-based application has called “Recover Me” tailors all unique needs of both the patient and the physiotherapist. The architecture of the mobile application is depicted in Fig. 8, which presents interactions between client-side (Flutter) and server-side (Firebase), showing the flows of data among key components.

The application is open for logging in for both users (patient and physiotherapist). First, for patient log-in, each patient should register, indicating his or her disease. Therefore, the patient selects a game from the list of games to engage with a VR environment. The games are chosen based on the patient’s preference. For each trail, a score is recorded, and then a summary of the total number of trials with a score index is given through a graph. Notably, before playing, a description of each game is associated. For the physiotherapist, a generated score for each patient indicates how s/he interacts with the game, showing an indication of therapy progress level. In a risky situation, which means failing to record scores in almost all games, the physiotherapist can interface with the patient, giving instructions to interact with. It is worth mentioning that the profile of each physiotherapist includes a photo, the specialization, and a WhatsApp connection.

### Artificial neural network model

The second stage of the rehabilitation system involved developing an artificial neural network (ANN) model to remotely predict the progress level of patients without the direct involvement of a physiotherapist. ANNs are computational models inspired by the structure and function of biological neural systems, consisting of numerous interconnected neurons (Saleh and Salaheldin 2022; Ye et al. 2024). The ANN was designed as a feedforward network with an input layer corresponding to game-derived features, two hidden layers (64 and 32 neurons, respectively) using ReLU activation, and a single output neuron with a linear activation function for regression. The model was trained using Mean Squared Error (MSE) as the loss function and the Adam optimizer with an initial learning rate of 0.001, a batch size of 32, and 100 epochs, incorporating early stopping to prevent overfitting. Hyperparameter tuning via grid search optimized the number of layers, neurons, activation functions, and learning rate. Regularization techniques, including dropout (0.2) and L2 regularization (0.01), were applied to enhance generalization. They are widely utilized in various applications, such as disease detection and diagnosis, due to their ability to map relationships between inputs and outputs through weighted connections, which represent the strength of these relationships (Cao et al. 2023). In this study, the ANN model was trained using the generated scores from each game, with regression techniques applied to account for the continuous nature of the training data. This approach enables ongoing monitoring and prediction of patient performance and progress levels. Consequently, the system allows patients to use the application independently without the need for real-time supervision by a physiotherapist.

### Experimental setup

The developed rehabilitation system utilizes VR settings to support the patient’s recovery process. A comfortable environment with adequate space for movement is essential for optimal engagement. To evaluate the system’s effectiveness, a four-week study was conducted involving 50 patients with various disorders. The participants included 28 females (56%) and 22 males (44%), with an average age of 41.2 years. Detailed participant information, including gender, age, diagnosis, initial assessment, and injury duration, is provided in Appendix 1. Initially, each patient completed six games, scoring out of 10, to assess their baseline skill level. The order of games was determined by the patient’s preference. Following this assessment, patients participated in a structured training program lasting four weeks, designed according to a physiotherapist’s guidance. Their performance was recorded weekly, and average scores from all training sessions were computed to derive a comprehensive overall score for each patient. Patients were allowed multiple attempts to achieve satisfactory outcomes for each game.

Several challenges were encountered by patients while engaging with the designed rehabilitation system. These included:

1. *Coordinating movements* Patients needed to align their physical movements with the system to effectively engage with the selected games.
2. *Adjusting eye movements* Synchronizing eye movements with the VR headset posed initial difficulties for some patients.
3. *Communication with physiotherapists* Ensuring effective communication to monitor performance and address concerns was essential during the sessions.

## Results

**Fig. 9:**
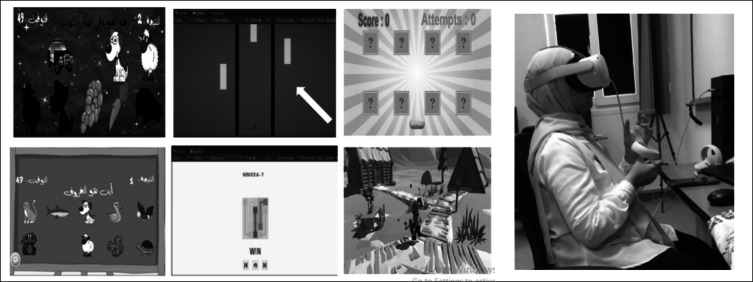
A real attempt at using the designed rehabilitation system for the upper limp based on the VR technology

**Fig. 10:**
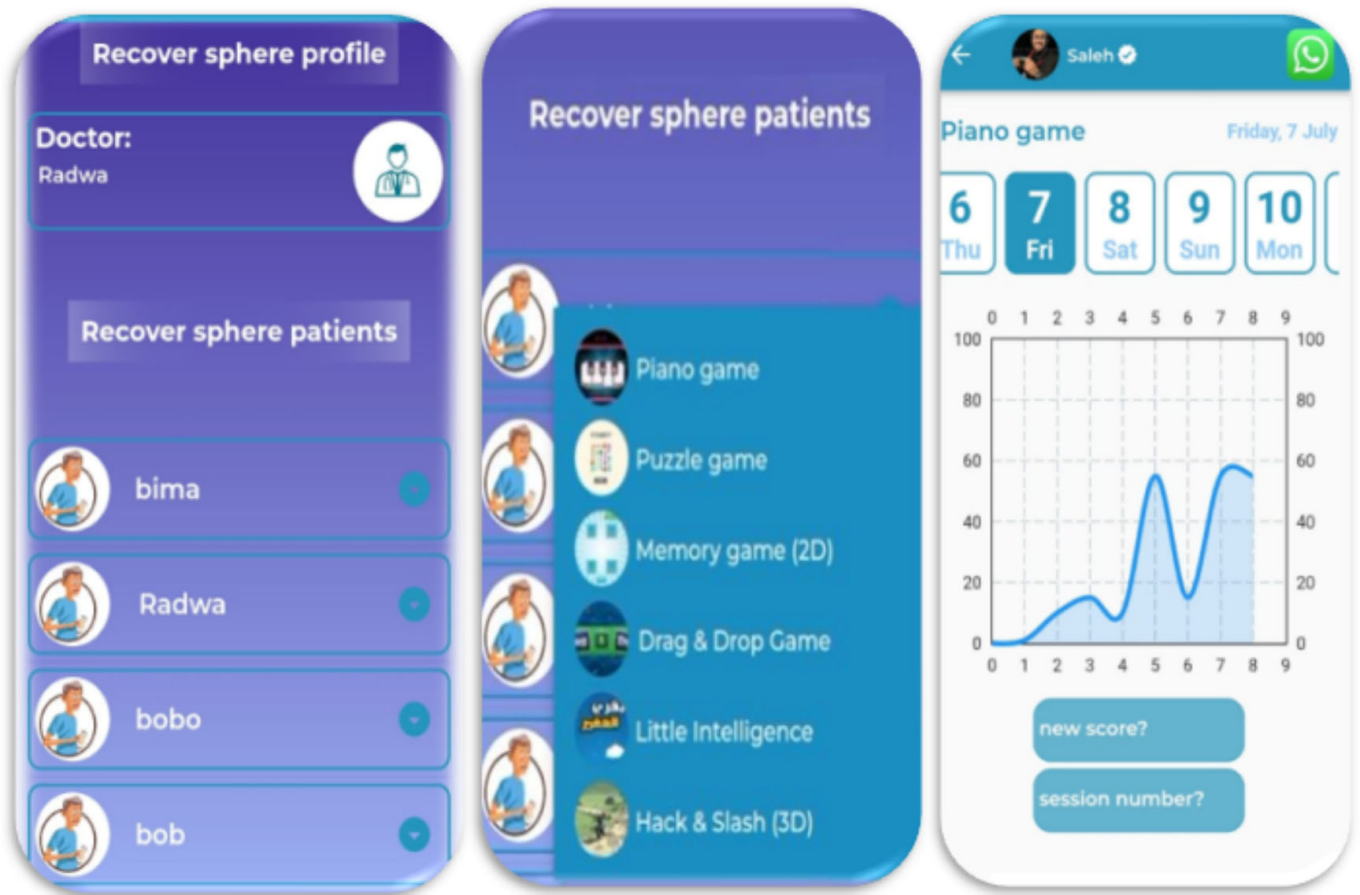
An example of a summary of “Recover Me” application indicating the patient, the physiotherapist, the games, and the total result of one game

A series of six games was designed to create a game-based rehabilitation system to alleviate the severity of neurological disorders. We designed all games to enhance upper limb movement and balance through engaging and interactive activities. Each patient should follow specific procedures, starting with logging into the system and ending with treatment progress follow-up. Table 1 summarizes the sequence of engaging with the games regarding the patient and the physiotherapist. Despite the three challenges mentioned earlier in the Experimental Setup section, the overall experience indicated that the VR rehabilitation system provided an engaging and effective approach to upper limb rehabilitation. Figure 9 illustrates patient interaction with the developed system, while Fig. 10 provides a summary of the “Recover Me” application.

**Table 1:** Sequence of gamification with designed games using the VR technology for rehabilitation of neurological disorders

Step no	Action	Description
1	Patient registration	After downloading the “Recover Me” application, each patient should register with basic information, including the type of disorder
2	Setting up the Oculus Quest 2 (VR headset)	Connecting the Oculus Quest 2 (VR headset) to the system following the instructions
3	Physiotherapist registration	The physiotherapist registers with the “Recover Me” app. for following up with each patient
4	Following game instructions	Concise instructions are provided by the physiotherapist explaining how to interact with a game
5	Playing games and tracking progress level	The patient selects game(s) according to the disease condition. A score index is given to the patient based on the attempts over time. Also, range of motion and balance status are indicated
6	Progress review	The physiotherapist reviews the performance of a patient through the generated score index. Additionally, he/she can communicate with the patient and give feedback
7	Follow-Up treatment plan	Based on the journey of gaming, the physiotherapist can assess the actual status and give recommendations for future treatment plans

The second phase of the rehabilitation system was to export the generated scores to the ANN model to train the network to estimate the progress level of each patient. In this context, we have 50 patients with various disorders played using the six games over four weeks. Many attempts were allowed for each patient according to his/her ability to record a series of scores out of 10. The best one was selected as an initial score for each game. Table 2 shows a sample of initial scores of 20 patients that were collected in addition to the total score percentage. Each game was indicated by the letter “G”. The dataset was divided into two parts: 80% (40 patients) for training the model and 20% (10 patients) for testing its accuracy, utilizing a fivefold cross-validation scheme.

**Table 2:** A sample of the collected data of five patients that were used in the developed rehabilitation system over four weeks

Week	Patient	G.1	G.2	G.3	G.4	G.5	G.6	Total %
1	1	9	2	3	2	8	2	43.33
	12	4	1	9	9	8	8	65.00
	25	6	2	7	4	7	4	50.00
	31	3	4	2	4	0	2	25.00
	38	8	2	4	1	4	6	41.66
2	1	5	5	2	4	7	6	48.33
	12	3	3	2	10	8	2	46.66
	25	10	9	2	5	7	2	58.33
	31	9	1	9	10	9	5	71.66
	38	9	3	0	2	2	0	26.66
3	1	7	4	4	6	5	0	43.33
	12	9	5	8	7	0	5	56.66
	25	5	9	4	7	4	6	58.33
	31	9	1	6	1	7	0	40.00
	38	10	8	6	0	7	5	60.00
4	1	2	3	2	8	9	4	46.66
	12	0	9	4	1	9	4	45.00
	25	5	1	9	6	7	4	53.33
	31	3	8	9	0	6	7	55.00
	38	1	6	4	0	6	6	38.33

The descriptive statistics as shown in Table 3 provide an overview of the dataset’s distribution for the Initial Assessment Score, Injury Duration, and Age. The mean Initial Assessment Score is 39.94, with a standard deviation of 16.21, indicating moderate variability, and scores range from 15 to 70, reflecting a widespread in the values. The mean Injury Duration is 10.74 months, with a relatively low standard deviation of 4.98, suggesting less variability compared to the assessment scores, and durations range from 3 to 18 months, indicating that most injuries lasted less than two years. The mean Age is 41.22 years, with a standard deviation of 6.54, showing moderate variability within a primarily middle-aged sample, ranging from 30 to 60 years. The low standard errors for all variables—2.29 for Initial Assessment Score, 0.70 for Injury Duration, and 0.93 for Age—indicate reliable and precise mean estimates. Notably, the Initial Assessment Score shows the highest variability, while Injury Duration has the lowest, suggesting greater consistency in injury periods across individuals.

**Table 3:** Descriptive statistics for the utilized dataset

Criteria	Initial assessment score	Injury duration (Months)	Age (Years)
Mean	39.94	10.74	41.22
Standard error	2.292028778	0.703814679	0.925507031
Standard deviation	16.20709091	4.976721321	6.54432298
Minimum	15	3	30
Maximum	70	18	60

**Fig. 11:**
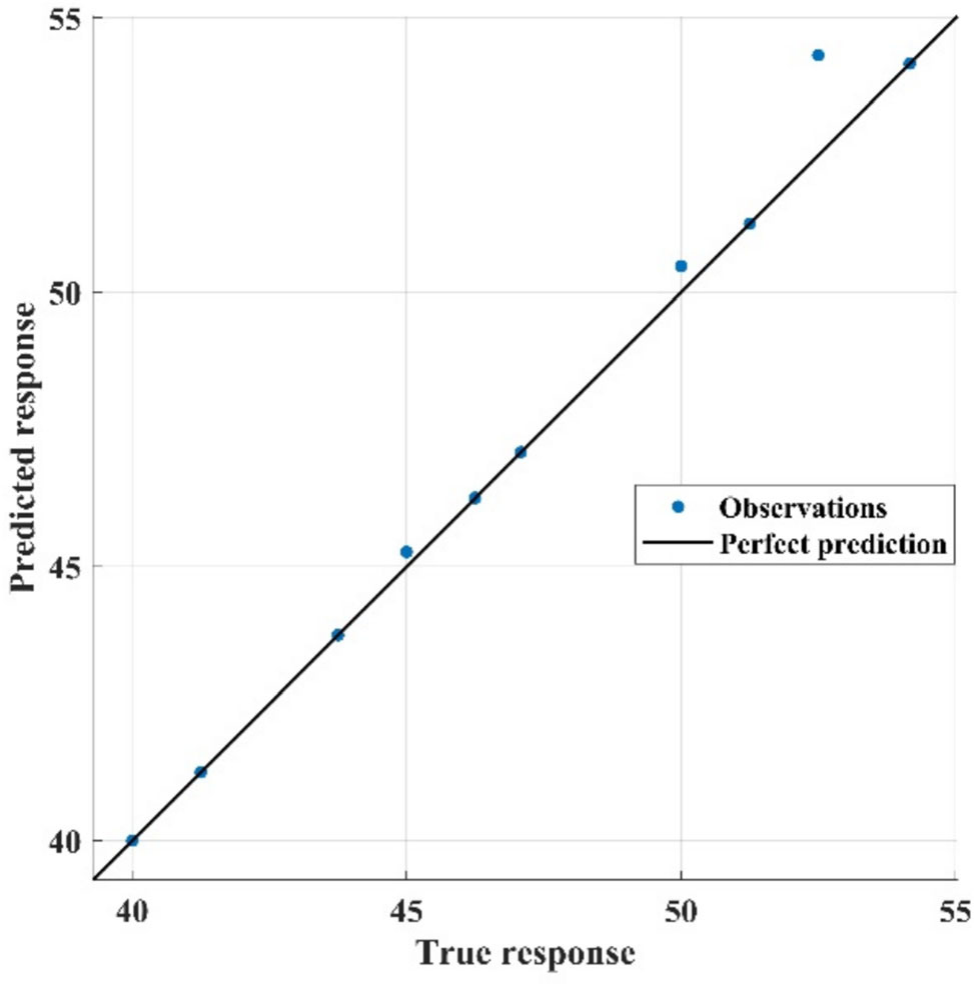
A relationship between the true scores and predicted scores based on the ANN model

**Fig. 12:**
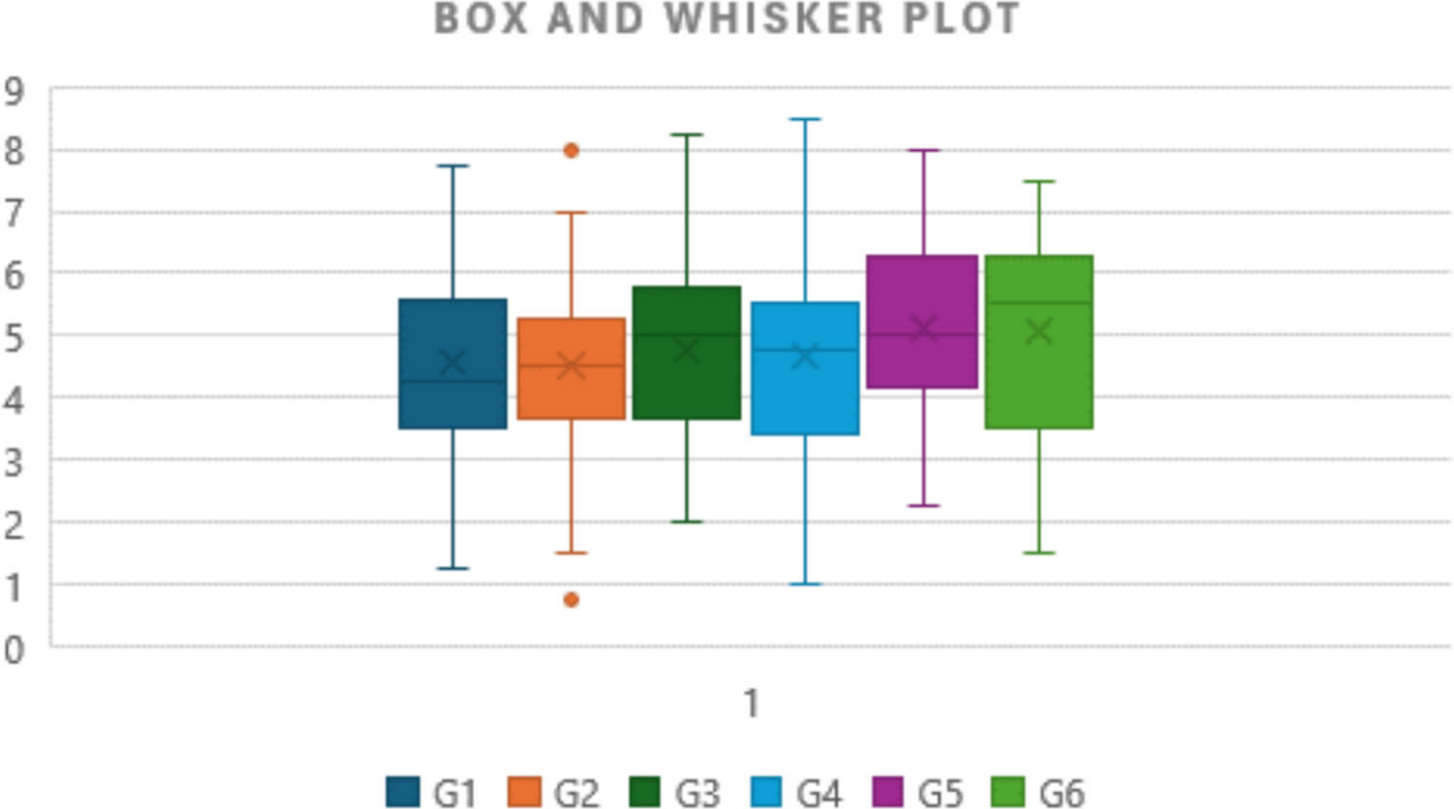
Box and whisker plot of the average scores over the four weeks playing with the developed six VR-based games

To validate the developed ANN model, we tested it on 10 patients (20%) of the data. The first week was selected as the baseline to measure the progress level. The results indicated that 60% of the patients showed positive progress, with improvements reaching up to 28.75%, as detailed in Table 4. Additionally, the regression analysis indicated a high correlation between the predicted value and actual value, as shown in Fig. 11. The analysis has recorded a correlation coefficient *R*^2^ of 0.98, mean square error (MSE) of 0.3599, and mean absolute error (MAE) of 0.2574. Notably, game 3 recorded the highest average score of 5.50 indicating its effectiveness among the other games. To present distribution of obtained scores, Fig. 12 depicts box and whisker plot of the average scores for the six games.

**Table 4:** Results of 10 patients indicating the progress level of rehabilitation using the developed game system

Patient	G1	G2	G3	G4	G5	G6	Week 1 score	Ture average score	Predicted score	Progress %
1	5.25	4.25	8.25	3.75	4.50	6.50	35.0000	54.1667	54.1652	19.17
2	1.75	4.75	5.00	3.25	7.00	6.00	51.6667	46.2500	46.2499	−5.42
3	5.50	3.00	5.25	3.25	5.75	5.50	40.0000	47.0833	47.0823	7.08
4	4.00	4.25	5.25	4.50	3.00	3.00	16.6667	40.0000	39.9975	23.33
5	3.50	5.00	5.75	1.75	4.50	4.25	41.6667	41.2500	41.2490	−0.42
6	4.25	4.75	7.25	6.75	2.25	6.25	50.0000	52.5000	54.3177	4.32
7	5.00	5.50	3.75	5.25	5.25	3.50	18.3333	47.0833	47.0823	28.75
8	5.50	6.75	4.75	2.75	2.75	7.50	41.6667	50.0000	50.4731	8.81
9	3.50	5.25	5.00	8.50	5.75	4.50	56.6667	54.1667	54.1652	−2.50
10	5.25	5.25	4.75	4.25	4.00	2.75	65.0000	43.7500	43.7484	−21.25

### External validation

To validate the designed rehabilitative system, an expert physiotherapist benchmarked the system against traditional physiotherapy plans for patients with underlying disorders. The expert tested the mobile application with ten patients, including five with stroke and five with cerebral palsy. Each patient tested the system, and their recorded scores were reported. The durations of their traditional physiotherapy plans were also recorded to assess the system’s effectiveness. Table 5 presents the results of the rehabilitation system, noting that the scores were obtained without prior training or repetition. Additionally, specific games were selected based on the disease, as detailed in Table 5. The results indicate that seven out of ten patients scored greater than 50%. According to the expert’s opinion, this is a promising approach that could lead to significant improvement if adopted consistently.

**Table 5:** Results of system validation with external ten patients with stroke and cerebral palsy

Patient	Gender	Age (years)	Disease	Injury duration (months)	G1	G2	G5	Mean
1	M	52	C. Palsy	5	4	6	8	6.00
2	M	64	Stroke	4	5	3	2	3.33
3	F	48	Stroke	6	7	5	4	5.33
4	M	44	C. Palsy	6	6	8	7	7.00
5	F	72	Stroke	5	4	5	3	4.00
6	F	58	C. Palsy	2	5	4	5	4.66
7	F	56	C. Palsy	8	8	5	5	6.00
8	M	62	Stroke	4	3	7	6	5.33
9	F	61	Stroke	10	8	9	5	7.33
10	F	57	C. Palsy	7	8	5	7	6.66

## Discussion

The developed game system has yielded results that demonstrate the effectiveness of the developed games in rehabilitating identified neurological disorders. Through the VR environment, any patient can play and interact with games. Each game was designed to simulate real-life scenarios where a patient can practice different activities related to the rehabilitation program. According to the physiotherapist who monitored the patients, almost all patients were not distributed to playing with the games. Indeed, provided instructions facilitated the interface with games. Besides, many attempts were made for each game to record an appropriate score. In this context, the mobile application “Recover Me” plays a pivotal role in monitoring and evaluating the patient and his/her status after applying the VR game system.

Concerning the goal of self-training and self-evaluating, we proposed the ANN model. It simulates the role of a physiotherapist in judging the performance and the progress level of a patient. A regression analysis has been carried out to evaluate the performance of the ANN model. Multiple parameters were used for this purpose, including *R*^2^, MSE, and MAE. For interpretation, a high correlation coefficient (0.98) means small residuals and a good fitness function. In contrast, small values of MSE and MAE mean a high accuracy of the regression analysis in predicting the scores. By applying the ANN model to ten patients, six of them have demonstrated improvements compared to their initial reference point (first week). While four patients have failed to record an improvement in their rehabilitation plan. In this context, further training could enhance utilization of the model. Moreover, prior knowledge of the benefits of gamification as a rehabilitation plan could promote patient motivation to achieve high scores.

Data distribution of average scoring index over four weeks as shown in Fig. 12 yielded skewness of the data. Only scores from game two demonstrated symmetric distribution; meanwhile, the other games scores depicted positive and negative skewness. Scores of games one and five achieved positive skewness (right-skewed), while scores of games three, four, and six demonstrated negative skewness (left-skewed). For all games, the median score recorded was between 4.5 and 5.5. Obviously, a wide distribution of scores has been recorded for game six, while a narrow distribution of scores has been recoded for game two. Therefore, game six achieved the highest recoded scores, and game two was vice versa. Explanations of differences between games are referred to as many reasons. First, the personnel aspects of the participants play a pivotal role in their practices. A few of the patients had never played video games. Second, some games, such as One and Five, demonstrated difficulty with the patients due to their type of impairment. Third, some games, such as “Drag & Drop,” need more playing time.

External validation of the system demonstrates its robustness. Although there was no prior training for the patients, a promising result has yielded. 70% of the patients succeeded in interacting with the system with an average score of 6.23. This implies system effectiveness even if a patient used to follow a traditional physiotherapy protocol. Ultimately, for tested data and external data, no risky situation has occurred. It means that almost all games have scored in all trails. Notable improvements are found comparing the initial assessment scores of the patients with those given in Table 2 for the fourth week. For example, the initial score of 35.00 for patient number 1 has improved to be 46.66 after the fourth week. This means an improvement of 33.3% has been recorded for patient 1. Similarly, 181.25% for patient 12, 233.3% for patient 25, 3.7% for patient 31, and finally 82.5% for patient 38 have been reported. As seen, a wide range of improvements have been made with the proposed system. This variation may refer to the type of disorder and the duration of injury. Besides, patient response alters among patients.

Comparing our rehabilitation system to the other systems demonstrating uniqueness and robustness of the presented system. The work presented in (Potigutsai and Sornil 2021) focused on hand and fingers disabilities. Additionally, the study used a deep learning algorithm (YOLO 3) to train 2D images for different positions hands. The goal was to evaluate the model in terms of effectiveness, enjoyment, and convenience. As seen, the patient categories were different and there was no mobile application to monitor and evaluate the patients through connecting with a physiotherapist. In addition, the VR environment was not used in this system. The cell phone AR settings were employed in another study through three serious games to rehabilitate stroke patients (Song et al. 2019). Patients’ rehabilitation results were assessed via a questionnaire, not with a mobile application as we developed. Also, only three games were developed in this study. Moreover, the developed games were tested on stroke patients; meanwhile, our study considered traumatic brain injury, cerebral palsy, spinal cord injury, and stroke patients.

Using a user-centred design approach, a rehabilitation system based on six games was created (Tamayo-Serrano et al. 2020). This system can also connect any patient with a physiotherapist through a telecommunication system, not via a mobile application as we developed. The gaming concept was based on interacting with a natural user interface module. It is a group of sensors that a patient wears to enable him/her to play the desired games. Therefore, the mean of interaction was different on our developed system. Although the VR technology has been used for rehabilitation in (Pham et al*.* 2015; Kantu et al. 2024), the methodologies and targets of rehabilitation were different. A webcam was used by Pham et al. (2015) to detect hand movement to monitor the rehabilitation progress of the hand. In Kantu et al*.* (Kantu et al. 2024), the system was designed as a robot to aid activities of daily life through six games as well. While our system was designed for home, Tamayo and Serrano (2020) have developed six game-based rehabilitation systems that can be adopted either from home or hospital. Unlike our study, the system was developed for stroke patients only. Detecting stress levels of obstetric brachial plexus injury, intellectual disabilities, and dyslexia was a different target of a physiotherapy plan (Vural et al. 2024). Two serious games were developed for those patients. Therefore, it targeted various ailments and had a different objective.

To the best of our knowledge and from the previous comparison, no relevant study has been conducted to assess patient performance and estimate the level of progress remotely. Additionally, developing a mobile application to communicate a patient with a physiotherapist was not discussed in previous literature.

Limitations of this study were relatively small numbers of patients involved in the gaming. The dataset was only gathered over four weeks. Even with the same number of patients, longer periods could result in more datasets. Additionally, prior knowledge of the benefits of gaming in achieving rehabilitation is relatively uncommon. As such, more efforts should be made for neurological disorders to understand the role of a game-based rehabilitation system in the treatment. Only six games were designed for rehabilitation. In this way, more games could participate in rehabilitating upper- limb disabilities.

## Conclusions

The VR game-based system has proven its effectiveness in rehabilitating neurological disorders and cognitive disabilities. Through six games, various types of patients can improve their abilities in upper-limb movements and thinking. Moreover, the mobile application allows patient monitoring and evaluation. According to the progress level, a physiotherapist can customize the gaming practices to achieve the best outcomes. The physiotherapist’s proactive engagement greatly enhanced the patient’s recovery process and yielded favourable results. Furthermore, we succeeded in gathering valuable data that supports the effectiveness of this system compared to conventional treatment methods. Many features characterized the developed system in terms of design and use. The system offers a cost-effective solution without compromising the quality of rehabilitation. The real-time feedback and interactive nature of the games have facilitated a more targeted and intensive rehabilitation experience, enabling patients to gain more progress in their rehabilitation. With its cost-effectiveness and potential for widespread accessibility, the VR game-based rehabilitation system has the potential to revolutionize the field of upper limb rehabilitation and significantly improve the lives of patients.

Future work involves designing additional games to address a wider range of targeted disorders. Collecting more datasets is essential to enhance the system’s effectiveness. These datasets can be used to train various AI algorithms, further improving the system’s outcomes. Additionally, integrating haptic feedback devices is recommended. These devices provide tactile sensations that mimic real-world interactions, enhancing the immersive experience for patients. Analyze patient movement patterns, speed, and accuracy to generate insightful reports that can guide treatment customization. A website platform could also facilitate the usage of the VR game-based system. Ultimately, this system can be generalized for other types of disabilities.

## Data Availability

The datasets generated during the current study are available from the corresponding author upon request.

## Appendix 1

See Table 6

**Table 6:** The utilized dataset in the study

Participant	Gender	Age (Years)	Diagnosis	Initial assessment score	Injury duration (Months)
P1	Male	33	C. Palsy	35	7
P2	Male	42	Stroke	51	7
P3	Female	48	Stroke	40	6
P4	Male	43	C. Palsy	16	13
P5	Female	60	Stroke	41	18
P6	Female	56	C. Palsy	50	18
P7	Female	47	C. Palsy	18	16
P8	Male	53	Stroke	41	14
P9	Female	41	Stroke	56	3
P10	Female	53	C. Palsy	65	11
P11	Male	43	Traumatic brain injury	43	8
P12	Female	44	Stroke	16	16
P13	Male	43	Spinal cord injury	15	16
P14	Male	36	Stroke	46	5
P15	Female	35	Spinal cord injury	29	6
P16	Female	47	Spinal cord injury	64	10
P17	Female	49	Stroke	39	8
P18	Female	40	Traumatic brain injury	24	4
P19	Male	31	C. Palsy	16	8
P20	Male	44	Stroke	22	16
P21	Female	42	Traumatic brain injury	47	3
P22	Male	36	Spinal cord injury	48	16
P23	Female	39	C. Palsy	21	5
P24	Male	43	Stroke	58	11
P25	Male	30	Traumatic brain injury	16	17
P26	Male	34	Spinal cord injury	54	18
P27	Female	35	Stroke	41	12
P28	Male	37	Spinal cord injury	23	18
P29	Male	36	Traumatic brain injury	36	6
P30	Female	42	Traumatic brain injury	44	14
P31	Male	40	C. Palsy	53	9
P32	Female	43	Stroke	70	9
P33	Male	31	C. Palsy	17	18
P34	Female	43	Traumatic brain injury	50	17
P35	Male	45	Spinal cord injury	59	6
P36	Female	41	Stroke	36	9
P37	Female	43	Spinal cord injury	67	4
P38	Female	45	C. Palsy	21	6
P39	Male	48	C. Palsy	59	4
P40	Female	36	Traumatic brain injury	41	12
P41	Female	33	Traumatic brain injury	51	16
P42	Female	30	Stroke	69	10
P43	Female	42	Spinal cord injury	20	18
P44	Female	39	Stroke	41	6
P45	Male	42	Stroke	52	11
P46	Female	47	C. Palsy	31	16
P47	Female	35	C. Palsy	43	8
P48	Male	39	Traumatic brain injury	39	11
P49	Male	40	C. Palsy	17	14
P50	Female	37	Traumatic brain injury	46	3

## Abbreviations

ADL: Activities of daily living
AI: Artificial intelligence
ANN: Artificial neural network
AR: Augmented reality
MAE: Mean absolute error
MSE: Mean square error
VR: Virtual reality

## References

[CR1] Barczak AM, Woźniak H (2019) ‘Comparative study on game engines’. Studia Informatica. Systems and Information Technology. Systemy i Technologie Informacyjne, (1–2)

[CR2] Cao Z et al (2023) Energy consumption of intermittent ventilation strategies of different air distribution modes for indoor pollutant removal. J Build Eng 69:106242

[CR3] Daoud MI et al (2020) A game-based rehabilitation system for upper-limb cerebral palsy: a feasibility study. Sensors 20(8):2416 32344557 10.3390/s20082416 PMC7219503

[CR4] Kantu NT, et al (2024) ‘Resist-as-needed ADL Training with SPINLDE for Patients with Tremor’. IEEE Trans Neural Syst Rehabil Engineering [Preprint] 10.1109/TNSRE.2024.3392615 38652620

[CR5] Lange B et al (2012) Designing informed game-based rehabilitation tasks leveraging advances in virtual reality. Disabil Rehabil 34(22):1863–1870 22494437 10.3109/09638288.2012.670029

[CR6] Oculus Quest 2 Specifications (2020). Available at: https://studiox.lib.rochester.edu/oculus-quest-2-specifications/ (Accessed: 5 January 2025)

[CR7] Pham NB, et al (2015) ‘Game-based virtual rehabilitation system for upper extremity using low-cost camera’. In 2015 8th Biomedical Engineering International Conference (BMEiCON). IEEE, pp. 1–5

[CR8] Potigutsai N, Sornil O (2021) ‘Hand and fingertip detection for game-based hand rehabilitation’. In 2021 IEEE International Conference on Big Data and Smart Computing (BigComp). IEEE, pp. 36–43

[CR9] Raymer E, MacDermott Á, Akinbi A (2023) Virtual reality forensics: Forensic analysis of Meta Quest 2. Forensic Sci Int: Digit Investig 47:301658

[CR10] Saleh N, Salaheldin AM (2022) A benchmarking platform for selecting optimal retinal diseases diagnosis model based on a multi-criteria decision-making approach. J Chin Inst Eng, Trans Chin Inst Eng, Series A 45(1):27–34. 10.1080/02533839.2021.1983466

[CR11] Singh S, Kaur, A (2022) ‘Game development using unity game engine’. In 2022 3rd International Conference on Computing, Analytics and Networks (ICAN). IEEE, pp. 1–6

[CR12] Sinpithakkul C et al (2018) ‘Game-based enhancement for rehabilitation based on action recognition using kinect’, in TENCON 2018–2018 IEEE Region 10 Conference. IEEE, pp. 303–308

[CR13] Song X, et al. (2019) ‘Cellphone augmented reality game-based rehabilitation for improving motor function and mental state after stroke’. In 2019 IEEE 16th International Conference on Wearable and Implantable Body Sensor Networks (BSN). IEEE, pp. 1–4

[CR14] Tamayo-Serrano P et al (2020) A game-based rehabilitation therapy for post-stroke patients: An approach for improving patient motivation and engagement. IEEE Syst, Man, Cybern Mag 6(4):54–62

[CR15] Vohera C, et al (2021) ‘Game engine architecture and comparative study of different game engines’. In 2021 12th International Conference on Computing Communication and Networking Technologies (ICCCNT). IEEE, pp. 1–6

[CR16] Vural ŞF et al (2024) Stress recognition from facial images in children during physiotherapy with serious games. Expert Syst Appl 238:121837

[CR17] Wang Y-W, Chen C-H, Lin Y-C (2020) ‘Balance Rehabilitation System for Parkinson’s Disease Patients based on Augmented Reality’. In 2020 IEEE Eurasia Conference on IOT, Communication and Engineering (ECICE). IEEE, pp. 191–194

[CR18] Wuang Y-P et al (2021) Effectiveness of kinesthetic game-based training system in children with visual-perceptual dysfunction. IEEE Access 9:153838–153849

[CR19] Ye J et al (2024) Optimizing the topology of convolutional neural network (CNN) and artificial neural network (ANN) for brain tumor diagnosis (BTD) through MRIs. Heliyon 10(16):e35083 39687857 10.1016/j.heliyon.2024.e35083 PMC11647943

[CR20] Yin L, Yu Y, Han F, Wang Q (2024) Unveiling serotonergic dysfunction of obsessive-compulsive disorder on prefrontal network dynamics: a computational perspective. Cereb Cortex. 10.1093/cercor/bhae258 38904079 10.1093/cercor/bhae258

[CR21] Yu Y, Wang H, Liu X, Wang Q (2024) Closed-loop transcranial electrical stimulation for inhibiting epileptic activity propagation: a whole-brain model study. Nonlinear Dyn 112(23):21369–21387

